# Transition from 2D to 3D SBA‐15 by High‐Temperature Fluoride Addition and its Impact on the Surface Reactivity Probed by Isopropanol Conversion

**DOI:** 10.1002/chem.202001646

**Published:** 2020-08-06

**Authors:** Maximilian Lamoth, Thomas Gries, Frank Girgsdies, Friedrich Seitz, Maike Hashagen, Frank Rosowski, Robert Schlögl, Elias Frei

**Affiliations:** ^1^ Department of Inorganic Chemistry Fritz Haber Institute of the Max Planck Society 14195 Berlin Germany; ^2^ BasCat—UniCat BASF Joint Lab Technical University Berlin 10623 Berlin Germany; ^3^ Process Research and Chemical Engineering, Process Catalysis Research BASF SE 67063 Ludwigshafen Germany; ^4^ Department Heterogeneous Reactions Max Planck Institute for, Chemical Energy Conversion 45470 Mülheim a.d.R. Germany

**Keywords:** 2D to 3D transition, FT-IR H/D exchange, high-temperature aging, mesoporous silica, NH_4_F addition, SBA-15

## Abstract

A systematic variation of the SBA‐15 synthesis conditions and their impact on the structural and chemical characteristics are reported. An incremental alteration of the hydrothermal aging temperature and time was used to induce changes of the highly ordered SBA‐15 structure. Any effects on the total surface area, mesopores size, micropore contributions, and pore connectivity are amplified by a combined incremental increase of the NH_4_F concentration. Based on changes of the unit‐cell parameter as a function of the mesopore size, and a feature in the low‐angle XRD pattern, useful descriptors for the disorder of the corresponding SBA‐15 are identified. An additional analysis of the Brunauer–Emmett–Teller (BET) surface area and pore size distributions enables investigations of the structural integrity of the material. This systematic approach allows the identification of coherencies between the evolution of physical SBA‐15 properties. The obtained correlations of the surface and structural characteristics allow the discrimination between highly ordered 2D SBA‐15, disordered 3D SBA‐15, and highly nonuniform silica fractions with mainly amorphous character. The fluoride‐induced disintegration of the silica structure under hydrothermal conditions was also verified by TEM. A direct influence of the structural adaption on the chemical properties of the surface was demonstrated by isopropanol conversion and H/D exchange monitored by FTIR analysis as sensitive probes for acid and redox active surface sites.

## Graphical abstract

**Into another dimension**: This systematic approach to the synthesis of SBA‐15 materials allows a direct connection between the synthesis conditions and the physical parameters. The comprehensive structural analysis highlights the impact of high‐temperature aging and NH_4_F addition on the structural transformations and changes of the pore structures, from 2D to 3D and finally to amorphous SiO_2_. The physical trends are extended to chemical ones by applying isopropanol conversion as a probe for the surface reactivity. Finally, the reactivities are explained by FTIR H/D exchange experiments.

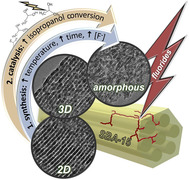

## Introduction

Mesoporous materials, especially mesoporous silica materials, are of great interest in a wide range of applications, such as molecular sieves, in catalysis, environmental analytics for adsorption and separation, or advanced optics.^1^, ^2^, ^3^, ^4^, ^5^, ^6^ They also exhibit a large structural diversity, leading to materials like MCM‐41^7^ consisting of unidirectional two‐dimensional hexagonal pores arranged in a honeycomb structure, HMS^8^ consisting of a wormhole framework structure, or KIT‐1^9^ offering a branched mesoporous channel network. The common synthesis strategy for such mesoporous silicas is based on a crystal templating approach, which strongly depends on the type and length of the surfactant, the alkaline character of the medium, the synthesis temperature, time, and washing procedure. Thereby, physical properties like surface area, pore sizes, or pore wall thicknesses are manipulated. An advanced version of the MCM‐41 materials is represented by SBA‐15,^10^ which exhibits a similar hexagonal mesoporous structure, but with larger pores and thicker pore walls. The improved pore wall thickness of this material significantly enhances the thermal and hydrothermal stability compared with mesoporous MCM‐41 and related silica,^11^, ^12^, ^13^ which makes it a suitable candidate for high‐temperature conditions that are potentially present for, for example, several catalytic reactions. Thus, SBA‐15, as a host and support material for metals like Cu, Au, and Ag, is applied as a catalytic active material. The corresponding supported or hosted metal nanoparticles show relevant catalytic activity in several reactions such as the oxidation of CO,^14^, ^15^, ^16^, ^17^, ^18^ propene,^19^ methanol,^20^ and cyclohexene,^21^ the reduction of nitrogen oxides,^22^ 4‐nitrophenol,^23^, ^24^ and hydrogen peroxide,^24^, ^25^ as well as A^3^‐coupling reactions of aldehydes, amines, and terminal alkynes.^26^

**Figure 1:**
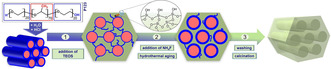
Overview of SBA‐15 synthesis starting with the formation of hexagonally arranged P123 rods (micelles) with a hydrophilic shell (PEO, blue) and a hydrophobic core (PPO, red). Upon TEOS addition (step 1), an amorphous silica network is formed around the rods, which is penetrated by hydrophilic tails of P123. These tails retreat upon hydrothermal aging (step 2), which can be supported by salt addition like NH_4_F. The SBA‐15 pre‐cursor is washed and calcined (step 3) to obtain a mesoporous silica SBA‐15.

The formation of metal nanoparticles inside the pores of SBA‐15 is beneficial owing to the good size, shape, and dispersion control based on its defined, long‐range ordered pore structures and tunable textural properties.^10^, ^27^, ^28^, ^29^ However, SBA‐15 materials have a strong tendency to form microporous channels and voids.^11^, ^30^ The microporosity is controlled by the nature of the surfactant, which is in the case of SBA‐15 the triblock co‐polymer P123. It consists of poly(ethylene oxide)‐poly(propylene oxide)‐poly(ethylene oxide) chains (PEO‐PPO‐PEO, see also Figure 1). Under standard synthesis conditions,^10^ micelles are formed where the hydrophilic PEO chains interact with each other sharing their hydration sphere. In addition, the EO headgroups of neighboring micelles also interact with each other. This interaction is still present when the silica network is formed, leading to microporous channels within the silica framework even after removing the template. This process is schematically represented in Figure 1.

The reduction of microporosity is based on the dehydration of the PEO chains and a decrease of the corresponding interaction. This causes a retreat of the PEO closer to the PPO part, which also leads to a swelling effect of the micelles, as illustrated in step 2 in Figure 1. To achieve this effect in practice, an increase of the synthesis temperature or hydrothermal aging at elevated temperatures can be applied.^11^, ^31^, ^32^, ^33^, ^34^, ^35^, ^36^, ^37^, ^38^

Another approach to manipulating the SBA‐15 properties is the addition of ionic compounds like fluorides, which influence, beside the hydrolysis rate of the silica precursor,^39^ the formation of the hydration sphere of the PEO chains. As a consequence, their interaction is weakened and the microporosity is reduced.^40^, ^41^, ^42^, ^43^, ^44^ Furthermore, fluoride is a well‐known catalyst for the polymerization of silica species.^45^, ^46^ It is proposed that the strong affinity of silicon to fluorine (and the corresponding ions) and the ability of F^−^ to attack Si^*x*+^ species leads to the expansion or substitution of its coordination sphere. Changing the Si coordination may facilitate the loss of ‐OR groups, generating more electrophilic species and consequently accelerates the polymerization.^47^, ^48^ The addition of NH_4_F as the fluoride source can further reduce the solubility of P123 in the aqueous solution based on a salt‐induced precipitation effect of both ionic species (NH_4_^+^ and F^−^), supporting the micelle formation.^49^

The addition of fluoride for the synthesis of mesoporous silica SBA‐15 is, in addition to the control of the microporosity,^40^, ^42^ also a common practice to manipulate the particle morphology (e.g., rods to sheets or platelets),^50^, ^51^, ^52^ particle size/length,^53^, ^54^, ^55^ and mesopore size.^28^, ^51^, ^54^, ^56^ However, it is important to note that fluoride addition combined with hydrothermal aging at elevated temperatures have been reported to lead to pore diameters close to (or even larger) than the distance between the pore centers and thus equal to the unit‐cell parameter. The corresponding wall thickness is commonly calculated by subtracting the mesopores diameter, derived from N_2_ physisorption analysis, from the unit‐cell dimension based on low‐angle X‐ray diffraction. In case of a mesopore diameter being larger than the unit‐cell dimension, the resulting apparent pore wall thickness will be negative.

It is very likely that the formation of interconnections between the mesopores, so called mesotunnels in the silica walls as described by Fan et al.,^57^ can be regarded as the origin of this apparent inconsistency, as the consequence is the overestimation of the mesopore sizes by N_2_ physisorption techniques. The authors named these kind of mesoporous silica “three‐dimensional (3D) SBA‐15” and observed their formation upon combination of hydrothermal treatment and the addition of tetramethylbenzidine (TMB; no fluoride source added). The assumption of an interconnected porous system is supported by Joo et al.^58^ for hexagonally ordered silica templated by polymers. Also, Kruk and Cao^56^ explained the appearance of a negative pore wall diameter by the presence of large mesoporous gaps in the pore walls, supported by the observation of a broadened pore size distribution. The addition of fluoride seems to support the interconnection, as Parfenov et al.^40^ reported that, although the microporosity was further reduced upon fluoride addition, the addition of high fluoride concentrations also prevented the formation of an ordered mesostructured SBA‐15 and, furthermore, promoted the appearance of negative pore wall diameters upon aging at 80 °C.

Although several reports^38^, ^58^, ^59^, ^60^ are leading to the conclusion that the applied temperature of the aging phase is the most important factor influencing the formation of the mesotunnels and 3D SBA‐15 materials, the influence of fluoride addition in combination with high‐temperature aging (HTA) is not well investigated yet. Very recently, Parfenov et al.^61^ investigated the structural consequences of fluorides used in the synthesis of SBA‐15 mesostructured silica with aging conditions at 80 °C. They reported a fluoride‐induced restructuring of the micropore system and the formation of coalesced micropores inside the silica wall. This is in good agreement with the observations already discussed for the application of high aging temperatures without fluoride addition.^56^, ^57^, ^58^

Within this study, we expand the so‐far reported investigations regarding the influence of fluoride, not only on fluoride addition at the standard aging temperature of 80 °C, but also combine the addition of varying concentrations of NH_4_F with HTA approaches of up to 140 °C. Thereby, we investigate the boundary cases of the transition from 2D to 3D SBA‐15 materials as well as the transition from 3D SBA‐15 to partially disordered mesoporous silica. The dimensionality thereby describes the character of the individual mesopores. As references, we also apply HTA without NH_4_F to clearly discriminate the contribution of thermal and fluoride‐induced effects. All synthesized samples were thoroughly analyzed by N_2_ physisorption techniques to identify Brunauer–Emmett–Teller (BET) surface area, microporosity, and pore size distributions. Changes in the unit‐cell parameter were identified by using low‐angle XRD. For the first time, the effect of fluoride addition during the synthesis of SBA‐15 materials is additionally correlated to their surface reactivity by using isopropanol conversion as a chemical probe, supported by FTIR H/D exchange experiments.

## Experimental Section

### SBA‐15 synthesis

Synthesis was divided into three categories. *Category I* is labeled as LTA (**l**ow‐**t**emperature **a**ging), *Category II* is labeled as HTA (**h**igh‐**t**emperature **a**ging), and *Category III* is labeled as HTAF_*X*_ (**h**igh‐**t**emperature **a**ging with **f**luorine), whereas *X* represents the corresponding NH_4_F equivalent added. In addition, all samples are labeled with its corresponding aging condition as *t*/*T*, with t=dwell time and T=temperature. For example, a Category III SBA‐15 material synthesized by using 0.05 equivalents of NH_4_F and aged at 110 °C for 24 h would be labeled as HTAF_0.05__24/110.

*Category I* is represented by the synthesis of conventional SBA‐15 without NH_4_F and aging at 80 °C. For this synthesis, an automated reactor setup (Mettler Toledo LabMax) was used, applying a constant stirring of 250 rpm. It was performed by first dissolving the triblock copolymer P123 (PEO_20_PPO_70_PEO_20_, 30 wt %, Sigma–Aldrich; 0.005 equiv) in 1.6 m HCl (Sigma–Aldrich, 37 %, diluted with ultrapure H_2_O; 0.005 equiv) at 45 °C followed by the addition of tetraethyl orthosilicate (Sigma–Aldrich, ≥99 %, TEOS; 1.000 equiv). The mixture was stirred for 1 day at 45 °C (precipitation phase) and subsequently aged at 80 °C for 12–84 h. Afterwards, the product was vacuum filtrated by using a P4 filter funnel and washed with the fourfold amount of ultrapure H_2_O. The filtered product was dried at 110 °C for 12 h and calcined at 550 °C for 6 h by applying a heating ramp of 1 °C min^−1^ under synthetic air.

*Category II* represents the synthesis without NH_4_F using aging temperatures greater than or equal to 80 °C. The educts P123, HCl, and TEOS were mixed and treated identically to the category I synthesis. After the precipitation phase, the mixture was transferred into a PTFE vessel (Berghof) and aged at a maximum temperature of 140 °C for varying times (see Table 1) by using a drying furnace (Haraeus T 5042). Subsequently, the material was filtered, washed, dried, and calcined as described in the category I synthesis.

**Table 1:** Overview of SBA‐15 synthesis conditions and properties.

Sample label	Aging *t* [h] and *T* [°C]	NH_4_F equivalents	*S*_total_ [m^2^ g^−1^]^[a]^	*S*_μ‐pore_ [m^2^ g^−1^]^[b]^	*D*_p_ [Å]^[c]^	*a*_0_ [Å]^[d]^	*a*_0_−*D*_p_ [Å]^[e]^
LTA_12/80	12/80	0	1053	373	73.1	111.0	37.9
LTA_24/80	24/80	0	992	283	75.9	111.9	36.0
LTA_84/80	84/80	0	955	277	81.4	115.0	33.6
HTA_24/80	24/80	0	962	300	75.9	111.6	35.7
HTA_24/100	24/100	0	1008	285	81.4	116.1	34.7
HTA_24/110	24/110	0	902	206	87.8	119.0	31.2
HTA_24/120	24/120	0	818	173	91.0	119.0	28.0
HTA_24/130	24/130	0	666	115	94.2	119.9	25.7
HTA_24/140	24/140	0	535	94	108.9	122.5	13.6
HTA_168/130	168/130	0	562	107	108.8	120.0	11.2
HTA_312/130	312/130	0	370	69	129.9	120.8	−9.1
HTAF_0.05__24/80	24/80	0.05	652	130	94.2	123.5	29.3
HTAF_0.10__24/80	24/80	0.10	542	93	104.9	124.8	19.9
HTAF_0.15__24/80	24/80	0.15	466	83	108.8	125.4	16.6
HTAF_0.20__24/80	24/80	0.20	426	75	116.8	125.2	8.4
HTAF_0.05__24/110	24/110	0.05	459	86	116.8	124.8	7.8
HTAF_0.10__24/110	24/110	0.10	523	62	113.0	125.2	12.2
HTAF_0.15__24/110	24/110	0.15	367	54	126.0	125.5	−0.5
HTAF_0.05__168/110	168/110	0.05	224	26	199.0	127.0	−72.0
HTAF_0.10__168/110	168/110	0.10	195	20	238.0	129.0	−109.0
HTAF_0.15__168/110	168/110	0.15	108	9	350.0	n/a	n/a
HTAF_0.05__24/130	24/130	0.05	470	75	104.9	121.9	17.0
HTAF_0.10__24/130	24/130	0.10	390	49	121.2	124.7	3.5
HTAF_0.20__24/130	24/130	0.20	230	24	129.9	126.7	−3.2
HTAF_0.25__24/130	24/130	0.25	194	10	166.9	127.0	−39.9
HTAF_0.05__168/130	168/130	0.05	191	13	166.8	125.5	−41.3
HTAF_0.10__168/130	168/130	0.10	154	13	199.0	125.9	−73.1
HTAF_0.20__168/130	168/130	0.20	26	4	273.7	125.8	−147.9

[a] Total BET surface area. [b] Micropore surface area. [c] Average pore diameter determined from the adsorption isotherms by the NLDFT method. [d] Hexagonal unit‐cell parameter determined from low‐angle XRD. [e] Apparent pore wall thickness estimated by subtracting the pore diameter value (*D*_p_) from the unit‐cell dimension (*a*_0_).

*Category III* represents the synthesis using aging temperatures greater than or equal to 80 °C and the addition of NH_4_F (0.05–0.25 equiv). The educts P123, HCl, and TEOS were mixed and treated identically to the category I synthesis. After the precipitation phase, the mixture was transferred into a polyethylene bottle into which the NH_4_F was added and further stirred for 10 min without heating. The NH_4_F‐containing mixture was subsequently transferred into a PTFE vessel (Berghof) and further treated as described for the category II synthesis.

### Low‐angle X‐ray diffraction

XRD patterns were recorded by using a conventional (i.e., wide angle) Stoe Stadi p transmission powder diffractometer, equipped with a primary focusing Ge monochromator (Cu_Kα1_ radiation) and scintillation counter. To enhance the accuracy of the 2*θ* scale, a measurement mode with two symmetric scans (negative and positive 2*θ*) was chosen. Small amounts of powdered sample were sandwiched between two layers of polyacetate film and fixed with a small amount of X‐ray amorphous grease. This sandwich was clamped into a sample holder ring. At low angles, small differences in 2*θ* result in significant errors on the *d*‐spacing scale. Misjudging the zero‐point on the 2*θ*‐scale by 0.01° results in a *d*‐spacing error of >1 %. Thus, the diffractions patterns were evaluated by using correlated fitting of the asymmetric diffraction peaks (see Figure S13 in the Supporting Information for fitting examples of two samples). An asymmetric instrumental function was convoluted with a symmetric Voigt function representing the sample contribution. A common lattice parameter *a* and a common 2*θ* offset (zero error) was refined on the available reflections of the two‐dimensional hexagonal lattice (10, 11, 20, 21, 30) for both scan ranges (negative and positive) simultaneously. Owing to the internal 2*θ* calibration based on the symmetric scan mode and correlated fitting, the instrumental zero error can be determined with high precision, yielding a more reliable determination of the *a*_0_ lattice parameter in turn. Thus, this procedure allows a robust and reproducible evaluation of the *d*‐values of differently treated samples. However, it needs to be kept in mind that both, owing to the asymmetric peak shape and the strongly asymmetric background, these values will depend strongly on the evaluation procedure applied. Thus, care should be taken when comparing the results of different studies on an absolute scale. As the absolute intensities of the reflections may vary both owing to progressive destruction of the pore ordering, and owing to some lack of reproducibility in the measurement (variation of sample amount and thickness, amount of grease applied), it was attempted to separate these effects at least qualitatively by the following scaling procedure. For comparative display purposes, the measured patterns were re‐scaled by normalizing to the fitted area (intensity) of the 10 reflection, thus giving a better impression of how the diffuse signal evolves compared with the reflections of the ordered pore system.

### N_2_ physisorption

Physisorption was performed with a Quantachrome Autosorb‐6‐b‐Mp after degassing a specific amount of the sample at 200 °C for 24 h by using a heating ramp of around 1 °C min^−1^. The resulting isotherms were recorded at the temperature of liquid nitrogen (−196 °C). Calculation of the surface area was performed according to the equation by Brunauer, Emmet, and Teller (BET equation).^62^ Therefore, adsorption data in the range *p*/*p*_0_=0.05–0.25 were used. The pore size distribution and average pore diameter were calculated from the adsorption isotherms by using nonlocal density functional theory (NLDFT; cylindrical pores, equilibrium model, for fitting errors see Table S1 in the Supporting Information).^63^ Micropores were analyzed in the range *p*/*p*_0_=0.20–0.40 by using the *t*‐plot method developed by De Boer et al.^64^

### Transmission electron microscopy

TEM imaging was performed by using a Thermo Fisher Scientific Talos F200X TEM operated at 200 kV acceleration voltage.

### Isopropanol conversion

Experiments was performed by using 1 mL of the catalysts in a sieve fraction of 200–300 μm, which was placed in a stainless‐steel plug flow reactor with 4 mm inner diameter. The samples were surrounded by quartz wool plugs on both sides. Prior to testing, all samples were pre‐treated at 300 °C for 3 h in synthetic air (21 % O_2_ in N_2_). The conversion test was performed by applying a heating ramp of 1 °C min^−1^ up to 250 °C followed by a dwell of 2 h at 250 °C. Isopropanol (Rotisolv ≥99.5 %, LC‐MS grade, Carl Roth) was filled into a gas saturator located before the reactor with a constant temperature of 21.9 °C (*p*_0_=0.0502 bar), which equals 1.2 vol % final isopropanol concentration in the gas feed, together with 5 vol % O_2_ and 93.8 vol % N_2_ at 100 mL min^−1^ overall gas flow. Oxygen is added to prevent the reduction of the silica surface, which suppresses the formation of isopropyl ether.^65^ Gases from and to the reactor and gas saturator flow through stainless‐steel tubes with inner diameters of 1/8 inches. Products were identified by using an online gas chromatograph (Agilent 6890 n) equipped with FID and TCD detectors.

### FTIR spectroscopy

Spectra were recorded by using a Varian 670 FTIR spectrometer equipped with a MCT detector. The spectra were recorded at a resolution of 2 cm^−1^, accumulating 16 scans. Self‐supported wafers (area weight of 4–6 mg cm^−2^, 10 mg cm^−2^ for Aerosil) were transferred into an IR cell connected to a vacuum line and a gas delivery system. Prior to the reaction, the catalysts were treated at 500 °C in vacuum for 4 h. After reducing the temperature to 400 °C, a reference spectrum was recorded followed by addition of 100 mbar D_2_. Spectra were recorded at 30 s intervals.

## Results and Discussion

An overview of all synthesized samples including their synthesis conditions and characterization data is presented in Table 1. In the following, SBA‐15 aging conditions are given as *t*/*T* (*t*=dwell time and *T*=temperature). Synthesis details are listed in the Experimental Section.

### High‐temperature aging and NH_4_F addition

Mesoporous silica SBA‐15, synthesized under standard conditions, is known for its high surface area of up to 1000 m^2^ g^−1^.^10^ These values are associated with a distinct microporosity, which might be undesired when SBA‐15 is applied as a support material for catalytic studies. A popular approach to reduce the microporosity is a high‐temperature aging (HTA) step, meaning the application of temperatures above 80 °C for defined aging times.^35^

As a reference for standard SBA‐15, a low‐temperature aging series (category I, LTA) at 80 °C was synthesized. Even with the LTA approach, the dwell time has a significant influence. Aging times of 12 h led to a total surface area (*S*_total_) of 1053 m^2^ g^−1^ with 373 m^2^ g^−1^ micropore surface area (*S*_μ‐pore_), whereas a doubling to 24 h led to a *S*_total_ of 992 m^2^ g^−1^ and *S*_μ‐pore_ of 283 m^2^ g^−1^. This corresponds to a reduction of the microporosity by approximately 25 %, whereas *S*_total_ was only reduced by approximately 6 %. A further increase of the aging time to 84 h led to a negligible change in *S*_total_ as well as *S*_μ‐pore_.

For the category II high‐temperature aging (HTA) samples, the aging temperature was modified. For this series, sample HTA_24/80 is a reproduction of LTA_24/80, differing in the use of Teflon pressure vessels for the HTA synthesis series. The repetition of HTA_24/80 in the modified setup led to a *S*_total_ of 962 m^2^ g^−1^ and *S*_μ‐pore_ of 300 m^2^ g^−1^, showing a reasonable agreement with the LTA approach. Therefore, it is stated that the modified setup has a negligible impact on the synthesis results. A clear correlation of *S*_total_, *S*_μ‐pore_, and the average mesopore diameter (*D*_p_) is identified for aging times of 24 h combined with temperatures of *T*=80–140 °C (Figure S1 in the Supporting Information). *S*_total_ decreases from 962 m^2^ g^−1^ (HTA_24/80) to 535 m^2^ g^−1^ (HTA_24/140), which corresponds to a loss of 46 %, leading to *S*_μ‐pore_ of less than 100 m^2^ g^−1^ (or a decrease by 69 %) with *D*_p_ increasing from 75.9 Å to 108.9 Å, all being in good agreement with the literature.^11^, ^35^ As mentioned above, these observations are explained by the retreat of the hydrophilic tails of the P123 into the mesopores and the accompanied swelling effect (Figure 1).

By determining the hexagonal unit‐cell parameter (*a*_0_) from low‐angle XRD, the apparent pore wall thicknesses can be estimated by subtracting *D*_p_ from *a*_0_. Hereby, this value steadily decreases from 35.7 Å for an aging temperature of 80 °C to 13.6 Å when increasing the aging temperature to 140 °C. Whereas for all 24 h HTA samples *D*_p_ increases with increasing temperature, *a*_0_ increases only for temperatures ranging from 80 °C to 110 °C and seems to reach a steady value between 119.0 Å and 119.9 Å for HTA samples treated at 24/110, 24/120, and 24/130. Such a transition of wall thickness and cell parameter of SBA‐15 might be the first indication for the formation of randomly distributed merged pores. This would also match with reported synthesis conditions for interconnected porous systems in SBA‐15 and the onset synthesis conditions for 3D SBA‐15 materials (dimensionality as description of the mesopore structure: interconnections between the 2D tube‐like pores are developing a 3D character).^38^, ^57^

Although aging temperatures of up to 200 °C are reported,^38^ an aging temperature of ≥140 °C has to be applied with care owing to the possible decomposition of P123.^11^ As the HTA_24/130 sample shows a lower proportional *S*_μ‐pore_ of 17 % compared with the 140 °C counterpart (HTA_24/140: 18 %), the increase in relative micropore surface area might derive from the template decomposition. Therefore, 130 °C is applied as the maximum aging temperature for SBA‐15 synthesis in the rest of this study.

**Figure 2:**
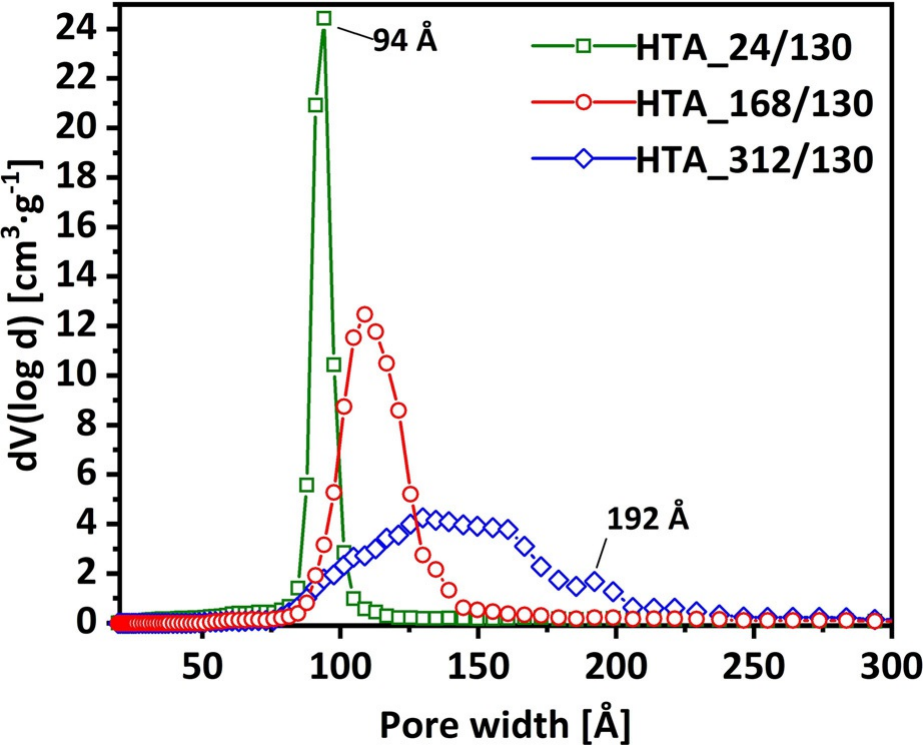
Pore size distributions of HTA samples aged at 130 *°C* for 24 h (green squares), 186 h (red circles), and 312 h (blue diamonds).

For the aging temperature of 130 °C, the dwell time was also varied from 24 h to 168 h and 312 h (samples HTA_168/130 and HTA_312/130, see Figure S2 in the Supporting Information). The strongest impact, comparing the 24 h dwell and the 312 h dwell at 130 °C, is detectable for *D*_p_, which shows a steady increase from 94.2 Å to 129.9 Å, whereas *a*_0_ is only slightly increased from 119.9 Å to 120.8 Å. This led to a decrease for the wall thickness from 25.7 Å (dwell: 24 h) to 11.2 Å (dwell: 168 h) to −9.1 Å (dwell: 312 h). The appearance of a negative pore wall thickness strongly indicates the formation of mesotunnels interconnecting the mesopores, leading to a 3D SBA‐15. The properties of sample HTA_312/130 is in very good agreement with the study of Yuan et al.^[38]^ in which 3D SBA‐15 was synthesized by using hydrothermal aging conditions of 190 °C for 24 h. Consequently, the increased dwell time (312 h vs. 24 h) potentially compensates for the use of the lower aging temperature (130 °C vs. 190 °C). This is supported by comparing the corresponding pore size distributions as shown in Figure 2, with the evolution of a broadened pore size distribution with increasing dwell time and the appearance of a second peak for pores with a diameter of 192 Å (sample HTA_312/130); this matches very well with the doubled average pore size of HTA_24/130 of 94.2 Å. These findings demonstrate the interconnection and the 3D porous network character for sample HTA_312/130. The increase of randomly distributed merged pores (relative to the ordered ones) with increasing temperature was already described in the literature and is in good agreement with the effect we observe for the increased dwell times.^[17]^

An additional SBA‐15 synthesis series was made by adding NH_4_F, which was added prior to the aging step (see Figure 1), labeled as HTAF_*X*_ (category III, high‐temperature aging with NH_4_F, *X*=equivalent NH_4_F). The stepwise increase of the NH_4_F concentration up to 0.20 equiv for 80 °C and 24 h aging conditions (Figure S3 in the Supporting Information) is accompanied by a pronounced decrease in *S*_total_ as well as *S*_μ‐pore_ down to 426 m^2^ g^−1^ and 75 m^2^ g^−1^, respectively, and an increase of *D*_p_ up to 116.8 Å (Figure S3 in the Supporting Information) with an almost constant *a*_0_. The corresponding wall thicknesses also decreased to 8.4 Å.

**Figure 3:**
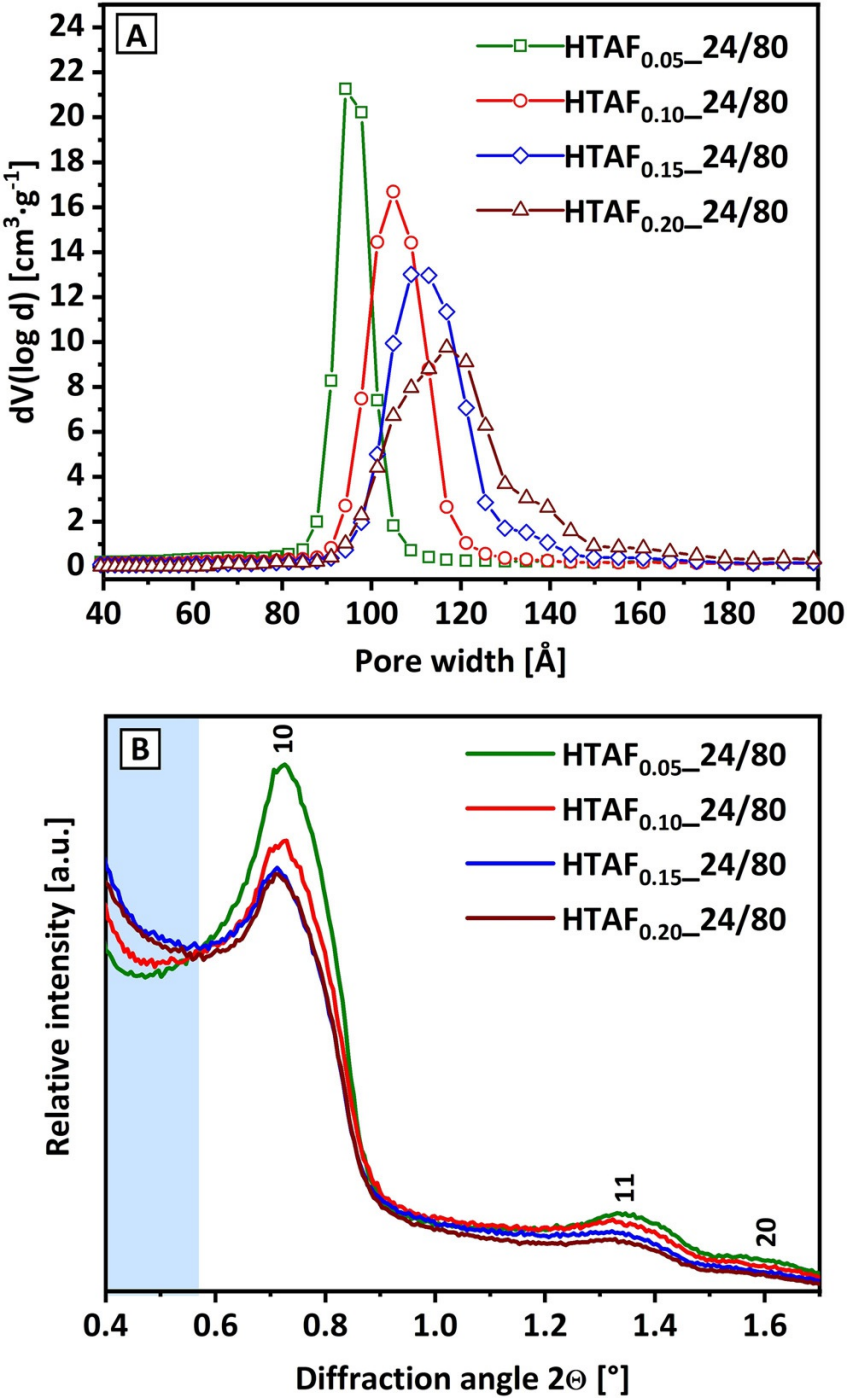
Pore size distributions (A) and low‐angle XRD (B) of samples aged at 80 °C for 24 h with the addition of 0.05 equiv NH_4_F (green), 0.10 equiv NH_4_F (red), 0.15 equiv NH_4_F (blue), and 0.20 equiv NH_4_F (wine). The blue highlight (B) shows the appearance of a small‐angle signal.

Parfenov et al.^61^ synthesized a comparable set of samples by using 0.025 equiv and 0.100 equiv NH_4_F added at the hydrothermal aging stage. The lower fluoride amount led to a sample without features of a 3D SBA‐15. The corresponding sample with the higher fluoride concentration is described by the authors as highly ordered with small amounts of micropores and also exhibiting criteria for a 3D SBA‐15 based on the nonuniform distribution of the silica wall density (electron density map calculated from experimental X‐ray pattern). Cell parameters as well as overall surface area of the described sample with the higher fluoride concentration of 0.100 equiv are in very good agreement with sample HTAF_0.10__24/80 (same synthesis conditions). Solely the pore diameter is divergent, which is likely based on the different applied methods for the determination of *D*_p_.^61^ Upon comparison of sample HTAF_0.10__24/80 with HTAF_0.05__24/80 (synthesized with less fluoride addition), the increased pore size distribution for HTAF_0.10__24/80 (Figure 3 A) and the small change in *a*_0_ in contrast to the significant change in *D*_p_ (see Table 1) matches with the described 3D SBA‐15 formation. Therefore, a transition from 2D to 3D SBA‐15 very likely occurs on increasing the NH_4_F concentration from 0.05 equiv to 0.10 equiv for aging conditions of 24 h at 80 °C.

Based on the identification of the first transition from 2D to 3D upon the addition of NH_4_F, an increasing fluoride concentration can be applied for ongoing investigations on the effect of increasing fractions of disordered/nonuniform pore channels (or the development of 3D porous networks). Figure 3 A illustrates the progressing development of the pore size distributions (maxima changing from 94 Å to 117 Å) for the HTAF series aged at 24 h at 80 °C with various NH_4_F concentrations (from 0.05 equiv to 0.20 equiv). The pronounced broadened pore size distribution with increasing NH_4_F additions leads finally to a second peak at around 140 Å (for 0.20 equiv), directly indicating the presence of mesoporous interconnections. This pore size at 140 Å seems already visible at 0.15 equiv NH_4_F (blue curve, Figure 3 A), which is interpreted as the starting point of the structural disorder.

The change in the SBA‐15 material can be further analyzed by low‐angle XRD. Based on this technique, a value for the unit‐cell parameter, *a*_0_, can be determined. In addition, the broadening of the corresponding reflection provides information on the relative amount of highly ordered arrangements present in a structure. A broadening of a reflection thereby indicates less contribution from ordered moieties. This means also the long‐range order of the material is decreased, also with consequences for reflections at higher 2*θ* values like the 11 and 20 reflections. Figure 3 B shows the low‐angle XRD analysis within the HTAF_*X*__24/80 series. The 10 reflections are equally broadened (intensity, that is, integral area of all 10 reflections are normalized to the same level). The decrease of the 11 and 20 reflections (relative to 10) indicate a loss of the long‐range order and less ordered mesopores.^66^ This stands representative for rather disordered and nonuniform structures, which is also supported by the results from the pore size distributions. In addition, a feature in the range of 0.4° to 0.65° is detectable in the low‐angle XRD pattern.

This signal shows an intensification with a loss in long‐range order (or with higher fluoride amounts). It is thereby reasonable that it phenomenologically indicates a loss of the highly periodic structure, that is, a decreased uniformity of the electron density of the pore walls as demonstrated by Parfenov et al.^61^ This hypothesis is supported by a matching decrease in the long‐range order by the progressing inhomogeneity of the pore size distributions and a reduction of the pore wall thicknesses, all indicating an accumulation of disordered structures. The small‐angle contribution in the low‐angle XRD in the range 0.4–0.65° will be further referred to as the “small‐angle signal”, directly connected to the loss in hexagonal order (or structural integrity) and increase of structural disorder. This is also reflected in the corresponding escalation of the pore width/distribution. This is in line with the pore size analysis of, for example, the HTAF_0.15_ sample (initialization of mesopores interconnection and increased structural disorder, Figure 3 A blue curve shoulder at 140 Å), where a significant increase in the “small angle contribution” is already visible (Figure 3 B, blue curve).

Interestingly, comparing the impact of temperature (HTA_24/130) and NH_4_F addition (HTAF_0.05__24/80), even a small portion of fluoride seems to have the same effect as a *T* increase by 50 °C, as almost identical values for *S*_total_, *D*_p_, and *a*_0_ are yielded. Also, upon comparing the pore size distribution (Figure S4 in the Supporting Information), it can be concluded that both samples are in good agreement with each other.

To better understand the effect of the NH_4_F addition on the SBA‐15 structure, the fluoride amount was varied at 110 °C and 130 °C aging temperature and aging times of 24 h and 168 h, respectively (Figure S5 and Figure S6 in the Supporting Information). As identified for the increased fluoride concentration, higher aging temperatures lead to the decrease of the *S*_μ‐pore_ (to 10 m^2^ g^−1^) and *S*_total_ (to 194 m^2^ g^−1^) for sample HTAF_0.25__24/130. However, also the wall thickness constantly decreases to −39.9 Å. The extended aging times from 24 h to 168 h lead to the same trends with a stronger decrease of all surface properties (*S*_μ‐pore_, *S*_total_) and an increase of *D*_p_, which stands for a pronounced negative wall thickness and a loss of the structural integrity (e.g., HTAF_0.20__168/130: −150 Å).

**Figure 4:**
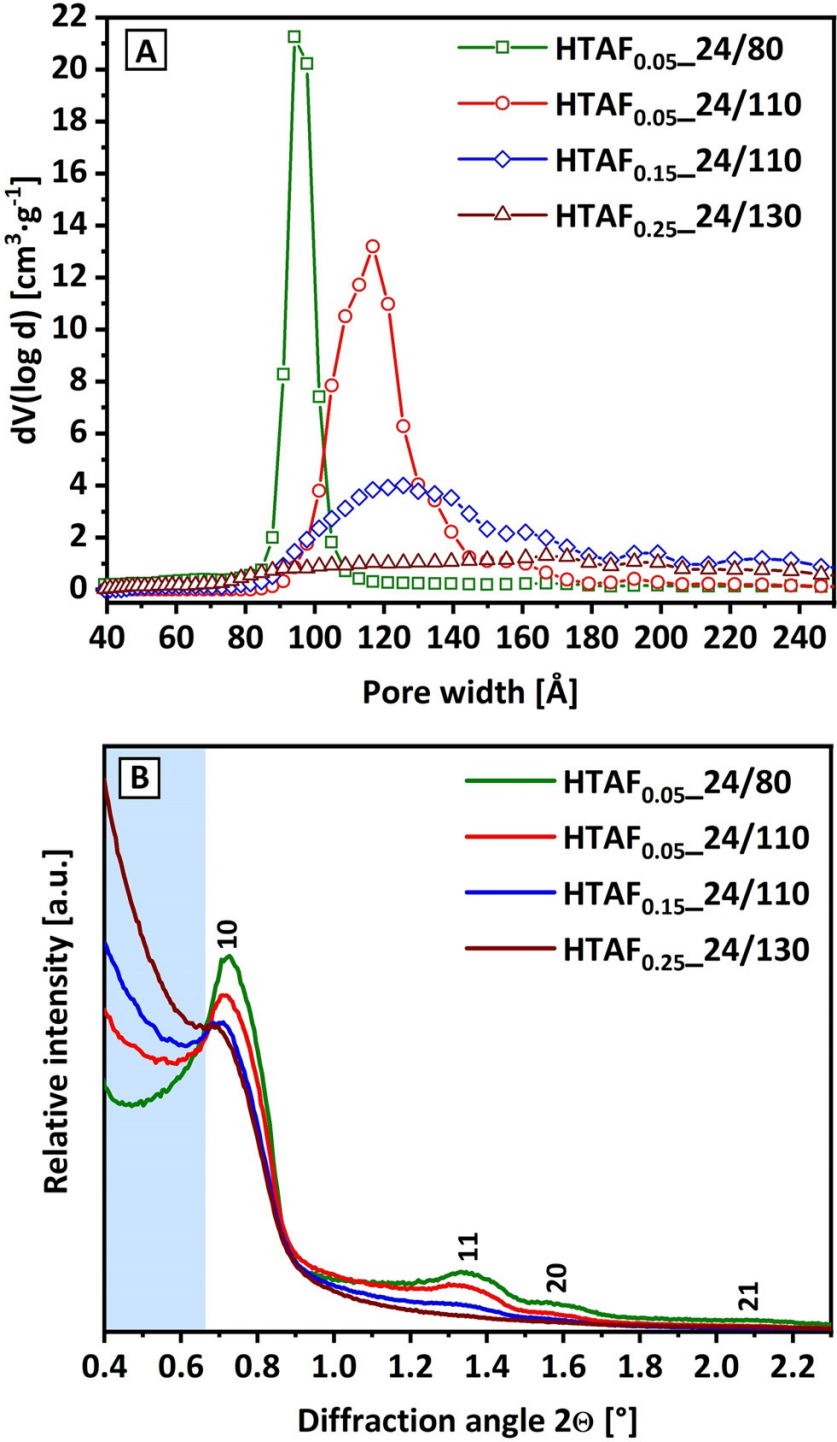
Pore size distributions (A) and low‐angle XRD (B) of samples aged for 24 h at 80 °C (green) and 110 °C with the addition of 0.05 equiv NH_4_F (red), 0.15 equiv NH_4_F at 110 °C (blue) and at 130 °C with the addition of 0.25 equiv NH_4_F (wine). The blue highlight (B) shows the appearance of a small‐angle signal.

For further analysis, the sample series of HTAF_0.05__24/80 to HTAF_0.05__24/110 (increasing aging temperature), to HTAF_0.15__24/110 (increasing fluoride content) to HTAF_0.25__24/130 (increasing fluoride content and aging temperature) are compared and illustrated in Figure 4 with respect to the change in pore size distribution and low‐angle XRD. An increasing fluoride concentration as well as an increased aging temperature cause a broadening of the pore size distribution and the development of additional peaks (Figure 4 A). This is interpreted as development of a 3D porous network. As analyzed for the HTAF_*X*__24/80 series, this is accompanied by a decreased long‐range order (Figure 4 B, decreasing 11 and 20 reflections) and an increase for the small‐angle signal (blue column, Figure 4 B). Consequently, the 2D character of the SBA‐15 materials gradually changes into 3D, whereby the order and uniformity of the sample is drastically reduced. This provides evidence for an interconnection of the mesopores finally leading to the loss of the SBA‐15 structure (i.e., no remaining pore structure and no structural order) towards an, for example, entirely amorphous silica. In addition, this is directly visible for sample HTAF_0.25__24/130, which is missing the characteristic 11 reflection in the low‐angle XRD pattern, indicating the loss of any long‐range order in the structure.

Regarding the size of the merged pores, sample HTAF_0.15__24/110 shows peaks at around 125 Å, 160 Å, 195 Å, and 230 Å. It represents an incremental increase of 30 to 35 Å per peak, clearly indicating that not only one kind of pores are interconnected but rather several types with different sizes. For even harsher conditions, for example, as applied for sample HTAF_0.15__168/110, the appearance of several peaks can be identified with an almost regular spacing of about 90 to 110 Å (Figure S7 in the Supporting Information). It matches with the interconnection of mesopores with a size comparable to the largest pores present for ordered 2D SBA‐15 materials (e.g., HTA_24/130 and HTAF_0.05__24/80 with ≈95 Å) and those at the early transition state from 2D to 3D SBA‐15 (e.g., HTAF_0.15__24/80 with ≈109 Å and HTAF_0.05__24/130 with ≈105 Å). Typically, these samples show several peaks, which further supports the irregularity of the pore interconnections.

**Figure 5:**
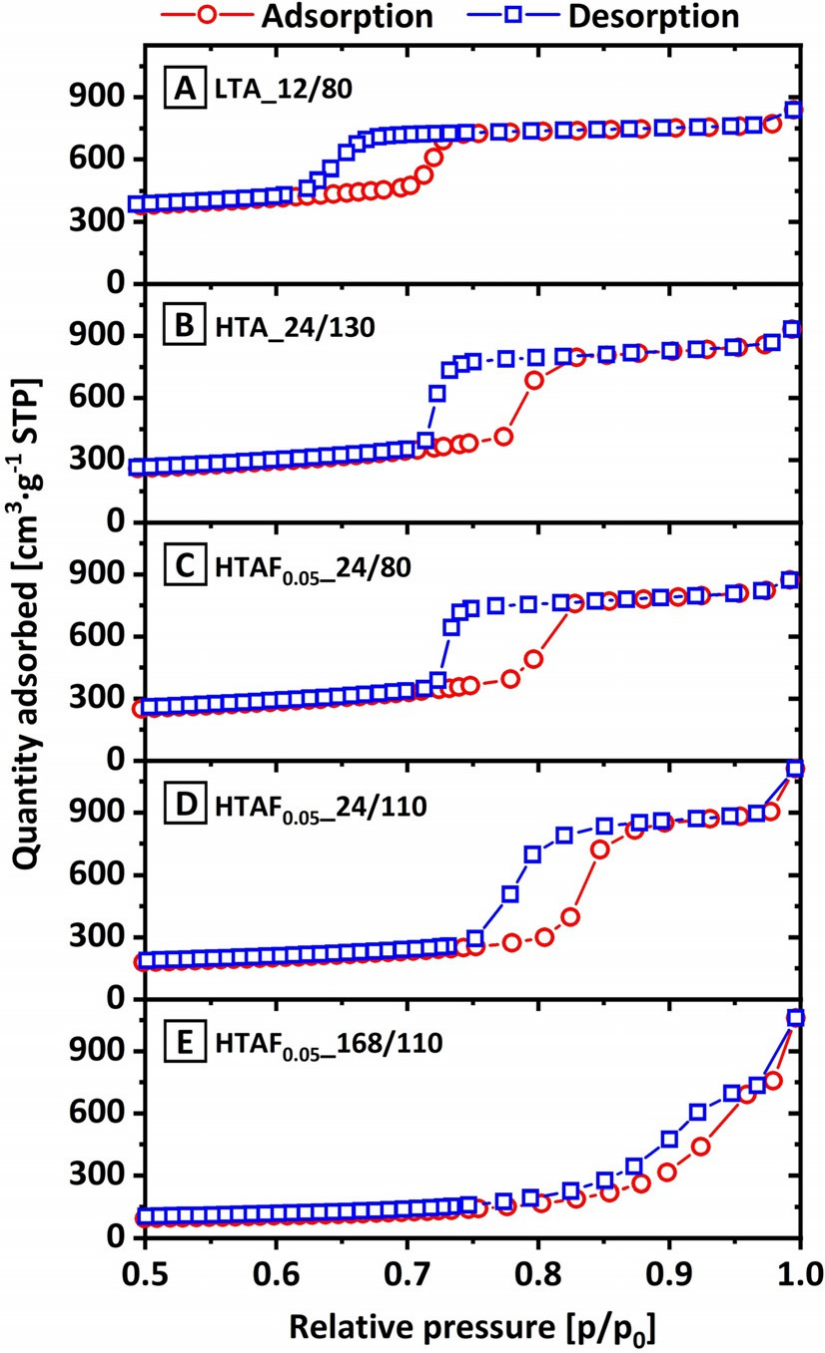
N_2_ physisorption isotherms with adsorption (circles, red) and desorption (squares, blue) for samples LTA_12/80 (A), LTA_24/80 (B), HTA_24/130 (C), HTAF_0.05__24/80 (D), HTAF_0.05__24/110 (E), and HTAF_0.05__168/110 (F). Full range isotherms are shown in Figure S8 in the Supporting Information.

Even the addition of small amounts of NH_4_F amplifies the effect of the HTA on the surface and pore properties. Coupled to longer dwell times, the influence is even more significant. To further investigate the different textural characteristics, the physisorption isotherms^67^ of sample LTA_12/80 (reference for standard synthesis conditions: highest micropore fraction and thickest pore wall diameter) is compared with a series of samples: HTA_24/130 (longer aging time), HTAF_0.05__24/80 (ordered 2D SBA‐15, NH_4_F addition), HTAF_0.05__24/110 (early transition 2D to 3D, NH_4_F addition, and higher temperature), and HTAF_0.05__168/110 (advanced transition 2D to 3D, NH_4_F addition, higher temperature, and longer time) as illustrated in Figure 5 and Figure S8 (in the Supporting Information).

For the reference sample (Figure 5 A), the adsorption and desorption isotherms show a type V isotherm with a H1 type hysteresis loop. This is typical for a narrow pore size distribution and uniformity of cylindrical pores, being typical for SBA‐15 silica. These structural properties also guarantee the good accessibility of the pores. The introduction of a high‐temperature aging step at 130 °C (Figure 5 B) as well as the addition of a small amount of NH_4_F at standard aging conditions (Figure 5 C) decreases the symmetry to a H2 hysteresis loop, which is usually explained by the formation of bottlenecks of relatively uniform channel‐like pores. An increase in aging temperature and dwell time strongly intensifies this effect. Thereby, sample HTAF_0.05__24/110 (Figure 5 D) shows first characteristics of a H3 type hysteresis loop, which is well developed for sample HTAF_0.05__168/110 (Figure 5 E). This kind of hysteresis loops are described for slit‐like pores with strongly irregular shapes and broad size distributions.^68^ Also, a shift to higher relative pressures for the hysteresis is observed, which indicates an increase of the pore sizes. These results are in very good agreement with the corresponding pore size distributions (Figure S9 A in the Supporting Information), low‐angle XRD analysis (Figure S9 B in the Supporting Information), and with the previous described effect for HTA treatments and fluoride addition.

For sample HTAF_0.05__168/110 (Figure 5 E), the characteristic mesoporous structure has almost vanished and additionally the absence of a plateau, as observed for the other samples at *p*/*p*_0_ larger than approximately 0.9, indicates the presence of even larger pores, which are not accessible by a standard N_2_ physisorption measurement. As described for sample HTAF_0.25__24/130, also sample HTAF_0.05__168/110 develops an amorphous character without a significant contribution from mesoporous structures.

**Figure 6:**
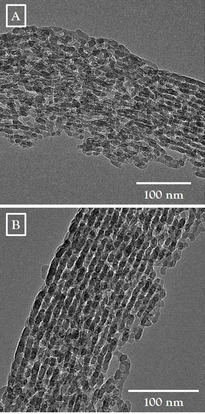
TEM analysis of sample HTAF_0.05__168/110 showing strongly disordered (A) and partially disordered structures (B).

For additional clarification of the structural motives, TEM analysis for sample HTAF_0.05__168/110, as shown in Figure 6 and Figure S10 (in the Supporting Information), was performed. The dominant motive is seen in Figure 6 A, with mainly disordered fragments of channel‐like structures without any order (long‐range order), and not well‐defined channels as shown in Figure 6 B. This is in line with our findings above and provides further evidence for the dominant amorphous structure with only a small remaining fraction of defined mesopores. Consequently, samples with *S*_total_ of <200 m^2^ g^−1^ might no longer resemble a mesoporous SBA‐15 structure. In parts, HTAF_0.05__168/110 also exhibits residual spots with well‐defined pore structures with diameters between 75 to 98 Å, which are in very good agreement with the dominant pore size for the reference sample LTA_12/80. This might by an indication that the NH_4_F addition accelerates the removal of micropores and the disorder of mesopores as a subsequent event, after forming the typical SBA‐15 structure. However, for those motives, breakthroughs between two channels are visible, which resembles the broad distribution of pore sizes and pore structures within the sample as already indicated by the determined pore size distribution.

**Figure 7:**
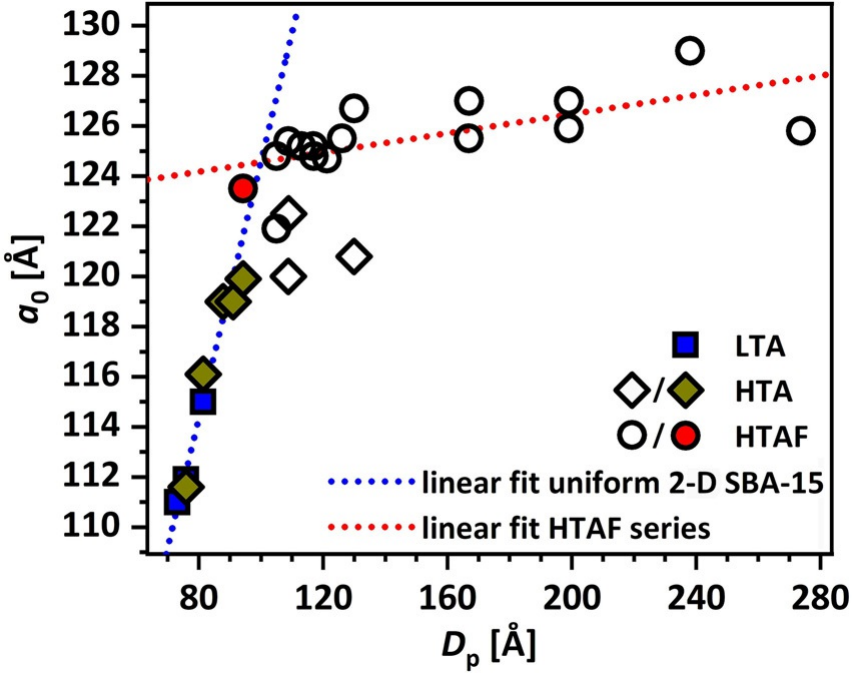
Unit‐cell parameter *a*_0_ as a function of *D*_p_ for series LTA (blue squares), HTA (empty and green diamonds), and HTAF (empty and red circles). Empty symbols indicate a nonuniform structure, filled symbols indicate a uniform pore structure.

Based on the described observations above, the small‐angle XRD signal seems to not only be a proxy for the structural disorder and the amount of non‐ordered structures (see trend, e.g., Figure 4 B), its absence might also be indicative for an intact 2D SBA‐15 structure. To clarify this, Figure 7 shows the correlation of the unit‐cell parameter *a*_0_ with the average pore diameter *D*_p_ for all synthesized samples in this work. For the LTA series, no small‐angle signal is visible, as well as for HTA samples aged at up 130 °C for 24 h and sample HTAF_0.05__24/80. The absence of mesotunnels and 3D features for those samples is also in good agreement with the literature.^35^, ^61^ For those 2D SBA‐15 samples (Figure 7, filled symbols), a linear correlation regarding the development of *a*_0_ as a function of *D*_p_ can be given (Figure 7, blue dotted line), which is expressed by the following equation [Eq. (1)]:

(1) $$a{}_{0}=73.25\pm 4.25\;+\;0.51\pm 0.05\;{}^{*}\;D{}_{p}$$

Samples that show a detectable small‐angle signal also show a higher value for *D*_p_ in relation to *a*_0_ based on Equation (1). A linear correlation for the HTAF series (Figure 7, red line) leads to an intercept with the 2D SBA‐15 linear correlation [Eq. (1)] at *a*_0_=124.6 Å and *D*_p_=99.9 Å. These SBA‐15 properties can be roughly regarded as a description for the transition of 2D to 3D SBA‐15 materials.

**Figure 8:**
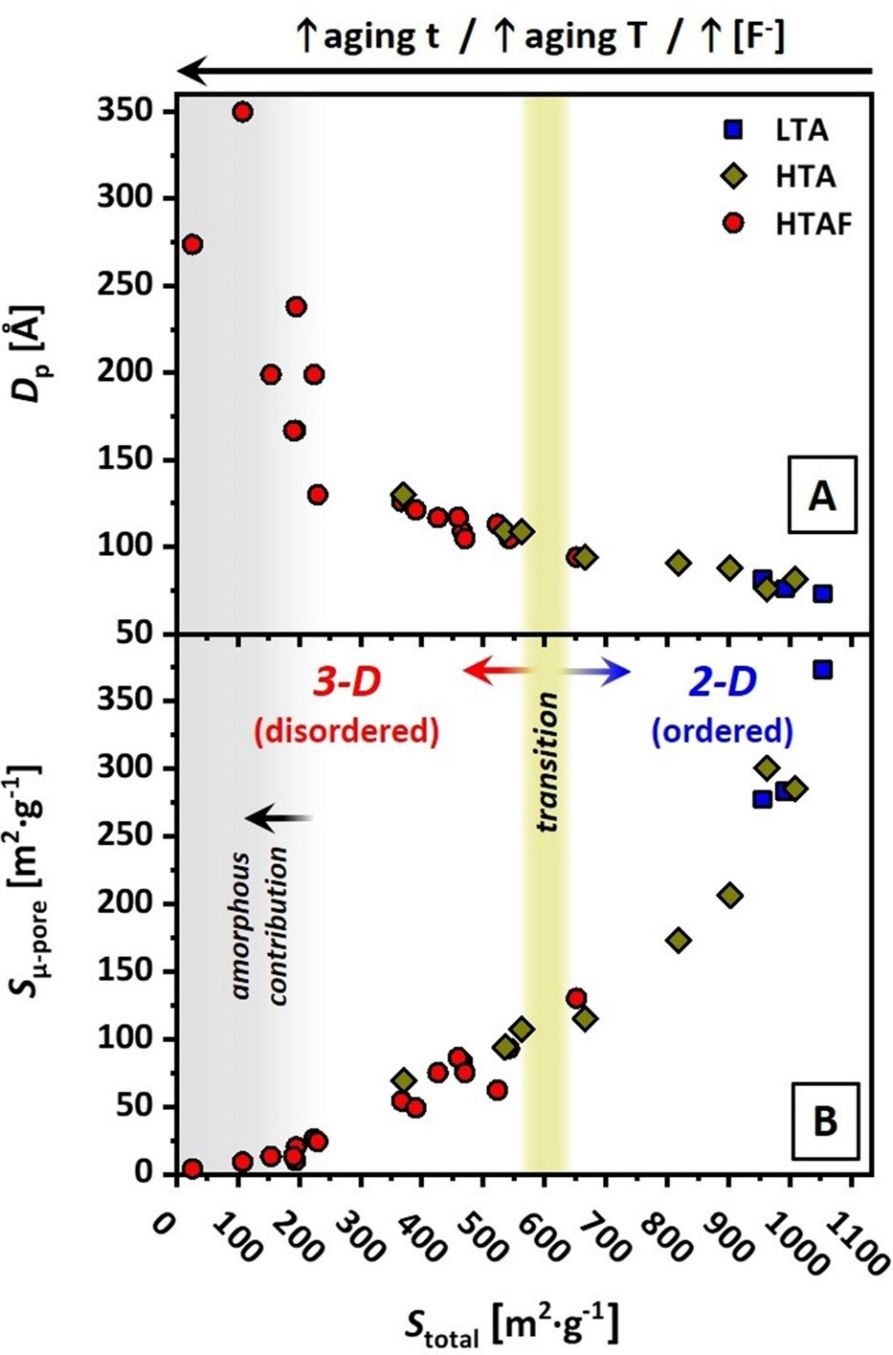
Mesopore diameters *D*_p_ (A) and micropore surface areas *S*_μ‐pore_ (B) as a function of the total surface area (*S*_total_) for all synthesized SBA‐15 samples using LTA (blue squares), HTA (yellow diamonds), and HTAF (red circles) aging approaches. The light‐green area represents the region for which the initial 3D pore networks (interconnections) are formed. For the gray region, a significant contribution from amorphous silica fractions is present.

In general, all aging parameters like time, temperature, and NH_4_F addition point in the same direction and might have an equal and cross‐correlating impact on all surfaces and structure parameters. In Figure 8, an overview of all synthesized samples is shown. Figure 8 A illustrates the *D*_p_ as a function of the *S*_total_. A rather linear correlation of *D*_p_ (between 125 Å and 75 Å) is yielded for samples in the range of *S*_total_ equal to 350–1050 m^2^ g^−1^. Below 250 m^2^ g^−1^, *D*_p_ is strongly enlarged and reaches values up to 300 Å, together with a significant amorphous SiO_2_ contribution. Figure 8 B compares the evolution of the *S*_μ‐pore_ to the corresponding *S*_total_. The decrease of *S*_total_ from 1050 to 800 m^2^ g^−1^ is accompanied by a very strong and exponential decrease in *S*_μ‐pore_ (370 to 170 m^2^ g^−1^). A rather linear correlation is reached for *S*_total_ in the range 700–200 m^2^ g^−1^ (*S*_μ‐pore_ down to ≈15 m^2^ g^−1^). Thereby, a certain contribution to the *S*_μ‐pore_ can also originate from interparticle voids, especially upon the pronounced disintegration of the ordered silica structure as observed for samples with *S*_total_<250 m^2^ g^−1^.

In this work, samples that exhibit a purely 2D character based on the given description [Eq. (1)] typically exhibit a *S*_total_ of larger than 650 m^2^ g^−1^, whereas samples developing initial 3D features were synthesized with a *S*_total_ of smaller than 570 m^2^ g^−1^. The clear correlation of the presented parameters enables the definition of an approximate transition from 2D to 3D SBA‐15 for samples with *S*_total_ of around 570–650 m^2^ g^−1^, with corresponding values for *S*_μ‐pore_ in the range 107–115 m^2^ g^−1^ and *D*_p_ of 104–94 Å. Based on this, the herein presented synthesis approach led to samples developing 3D SBA‐15 characteristics if *S*_total_<570 m^2^ g^−1^, *S*_μ‐pore_<107 m^2^ g^−1^, and *D*_p_>104 Å. The corresponding values for *a*_0_ for a purely 2D SBA‐15 are decoupled and described by Eq. (1) up to *a*_0_≈125 Å.

As analyzed by low‐angle XRD, TEM, and physisorption isotherms, the development of mesotunnels, leading to the 3D character of the mesopores (‐tunnels), also effects a decrease from highly ordered states with a symmetrical hexagonal structure into the formation of disordered and nonuniform fractions. The promotion of this effect by higher fluoride concentrations, higher aging temperatures, and longer aging dwell times is coupled to an increasing amount of fractions exhibiting no structural order and therefore are regarded as amorphous (low‐angle XRD Figure 4 B at <0.65°, TEM analysis Figure 6 B and Figure S10 in the Supporting Information). This fraction is analyzed to be significant for samples with *S*_total_<250 m^2^ g^−1^.

### Isopropanol conversion

To translate the described physical and structural trends into chemical ones, selected SBA‐15 samples were tested in the isopropanol conversion reaction under oxidative atmosphere (O_2_/C_3_H_8_O=1:0.24).^69^ This reaction was applied as a suitable probe for the surface related reactivity and the functionalization of specific sites (acid and/or redox active), respectively (normalized to the surface area of the SiO_2_ materials). This chemical analysis allows a correlation of the nature of the available surface, coupled to disordering effects and structural integrity as highly important parameters with respect to their application as, for example, support materials for catalytic reactions.^70^

**Figure 9:**
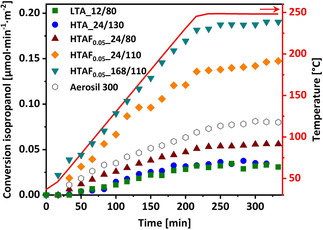
Overview of isopropanol conversion tests for samples LTA_12/80 (green), HTA_24/130 (blue), and samples with NH_4_F addition of 0.05 equiv treated at 24/80 (wine), 24/110 (orange), and 72/110 (dark cyan). In addition, commercially available fumed silica Aerosil is tested as a reference (gray).

The overview of the selected samples tested in the isopropanol conversion, including a reference sample of a commercially available, amorphous, and nonporous hydrophilic fumed silica (Aerosil® 300, Degussa, *S*_total_=328 m^2^ g^−1^), is shown in Figure 9 and the details are listed in Table 2. Hydrophilic fumed silica was synthesized by using very high temperatures larger than 1700 °C^71^ and therefore it exhibits a high chemical inertness, making it a suitable reference for a low chemical surface reactivity sample for the isopropanol conversion reaction. The reactivity is given in μmol per minute per surface area of the silica sample as a function of the testing time. After heating to 250 °C, all samples were dwelled for 2 h at 250 °C.

**Table 2:** Overview of average isopropanol conversion activity for a 2 h dwell at 250 °C, the corresponding relative conversion and relative OH group density (IR spectrum, Figure S11 in the Supporting Information) based on the Aerosil reference.

Sample	Conv. isoprop. [μmol min^−1^ m^−2^]	Relative conv. isoprop.	Relative OH group density
LTA_12/80	0.034	0.43	0.99
HTA_24/130	0.036	0.46	0.81
HTAF_0.05__24/80	0.055	0.70	1.08
HTAF_0.05__24/110	0.143	1.81	0.92
HTAF_0.05__168/110	0.189	2.39	0.93
Aerosil 300	0.079	1	1

The lowest activity in isopropanol conversion is observed for the LTA_12/80 and HTA_24/130 samples. These samples represent, within the series of highly ordered 2D SBA‐15 materials, the boundary cases of high (LTA_12/80: 1053 m^2^ g^−1^) and low (HTA_24/130: 666 m^2^ g^−1^) *S*_total_. The resulting low activities (Figure 9, green squares and blue spheres, respectively) confirm the structural integrity of the HTA_24/130 sample, already indicated by the *D*_p_ (94.2 Å) and *a*_0_ (119.9 Å) values interpreted as structurally intact 2D SBA‐15.

As the NH_4_F addition has a significant influence on the physical properties, three samples with an NH_4_F addition of 0.05 equiv were additionally tested in the isopropanol conversion reaction. These samples differ in aging temperature (HTAF_0.05__24/80 and HTAF_0.05__24/110) or dwell time (HTAF_0.05__168/110) and exhibit a higher conversion compared with the previously described samples (gradually increasing with increasing aging time and temperature, Figure 9, dark‐red pyramids, orange diamonds, and turquoise triangles). For sample HTAF_0.05__24/80, the conversion is increased by a factor of 1.6 (average conversion during the dwell time at 250 °C vs. LTA_12/80, see Table 2). This seems to be somewhat surprising, as this sample is regarded as intact 2D SBA‐15 (according to low‐angle XRD). However, low‐angle XRD is a rather bulk‐sensitive method, and the surface‐sensitive isopropanol conversion serves as complementary tool, identifying changes in the sample's chemical surface structure. As this SBA‐15 HTAF_0.05__24/80 still exhibits a lower activity than the reference silica Aerosil 300, a comparably low chemical surface reactivity is evidenced.

The relatively high reactivity for samples HTAF_0.05__24/110 and HTAF_0.05__168/110 (see Table 2) is in line with the findings of an elevated loss in highly ordered structures and confirms an ongoing surface functionalization of the SBA‐15 material. In addition, it supports the hypothesis that the removal of micropores (and loss in structured mesopores) by NH_4_F is a subsequent event, following the 2D SBA‐15 formation. The average conversion for, for example, sample HTAF_0.05__168/110 is 5.6 times higher as for the LTA_12/80 sample. Interestingly, the pore distribution itself seems to be a negligible factor in terms of isopropanol conversion, as it is independent of the micropore surface area of 373 m^2^ g^−1^ (LTA_12/80, 35 % of available surface area), or 115 m^2^ g^−1^ (HTA_24/130, 17 % of available surface area), the surface area based activities are unaffected.

In summary, the isopropanol conversion results are interpreted as a suitable chemical probe, identifying complementary information as a bridge to the physical and structural investigations. Only a combination of the applied techniques provides a reliable set of information, needed to judge the structural integrity of SBA‐15 materials.

### FTIR investigation of surface OH groups

A change in the isopropanol conversion reaction can have multiple origins, however, hydroxyl functions (OH groups) are, very likely, the most important candidates. To determine the number and nature of OH groups on selected silica samples, various FTIR measurements have been conducted.

After a thermal pretreatment (500 °C in vacuum for 4 h), all samples display a sharp band at 3739 cm^−1^ with a pronounced tail on the lower wavenumber side (Figure S11 in the Supporting Information). Both the peak position and small FWHM of the sharp peak are characteristic of free (i.e., non‐hydrogen‐bonded) OH groups.^72^ The tail indicates the existence of several types of hydrogen‐bonded OH groups.^72^ Under the assumption that the extinction coefficients are identical for all investigated samples (they might still differ between various types of OH groups), a relative concentration of surface OH groups is calculated (Table 2; integrated absorbance, normalized to area weight of the samples, Figure S11 in the Supporting Information, and the BET surface areas, Table 1). As there is no significant difference between the SBA‐15 samples and no trend following the specific synthesis conditions (i.e., increasing temperature, dwell time and/or increasing fluoride concentration), the OH density (number of OH groups) cannot explain the different reactivities in the isopropanol conversion.

**Figure 10:**
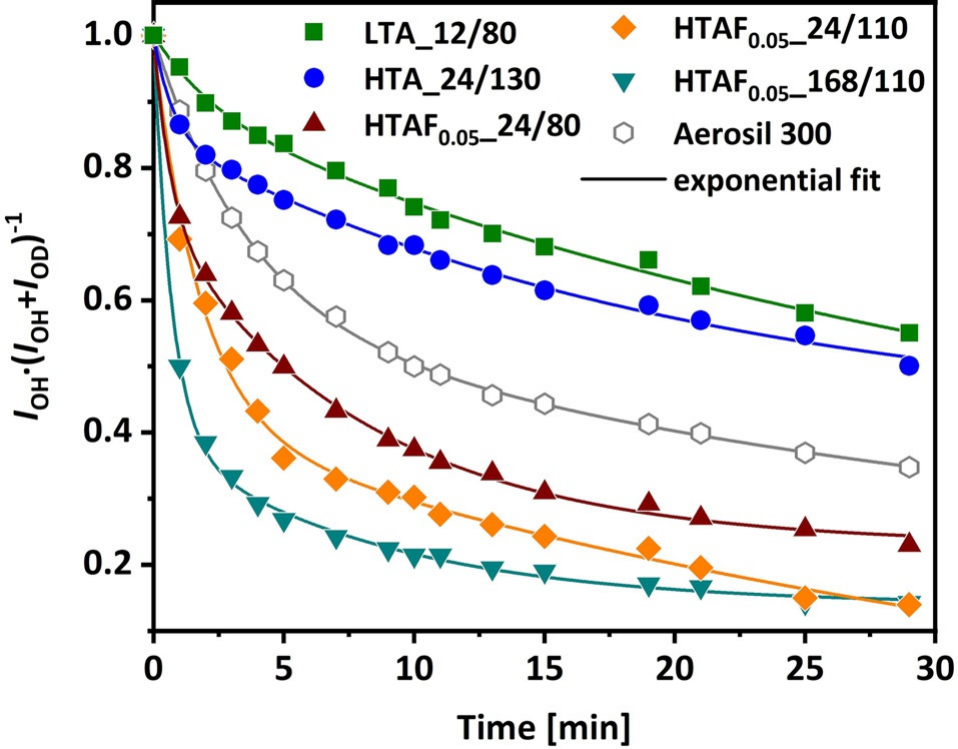
H/D exchange at 673 K for samples LTA_12/80 (green), HTA_24/130 (blue), and samples with NH_4_F addition of 0.05 equiv treated at 24/80 (wine), 24/110 (orange), and 72/110 (dark cyan). In addition, commercially available fumed silica Aerosil is shown as a reference (gray). The corresponding lines are a guide to the eye.

It has been shown that isotopic exchange with deuterium allows us to distinguish OH groups in zeolites.^73^ Therefore, different SBA‐15 types were exposed to 100 mbar D_2_ at 400 °C. As a consequence, a decline of the OH bands with a concomitant formation of an OD band at 2754 cm^−1^ is observed (Figure S12 in the Supporting Information). A significant difference in the reaction rates between the different SBA‐15 types, as illustrated in Figure 10, is observed. Comparing the synthesis conditions, SBA‐15 samples with an increased temperature, dwell time, and/or increased fluoride concentration show a faster and more pronounced H/D exchange. This trend follows the same order of reactivity as observed for the isopropanol conversion. Furthermore, the different kinetic profiles (different exponential fits) point to the existence of various types of OH groups with a different reactivity towards D_2_.

Novakova et al.^73^ postulated that Lewis acid sites are a prerequisite for H/D exchange in zeolites, whereas OH groups have little impact. Consequently, the most likely explanation for differing isopropanol conversion activities is the presence of different Lewis acidic sites for the silica samples, originating from the varying synthesis strategies. The reactivity in the H/D exchange is thereby in very good agreement with the corresponding isopropanol conversion data, evidencing a correlation of the increasing disorder of the SBA‐15 structure and the nature of the Lewis acid sites. This is also in line with the previously discussed results from, for example, low‐angle XRD and N_2_ physisorption techniques.

## Conclusion

The iterative variation of hydrothermal aging conditions and NH_4_F addition for SBA‐15 synthesis conditions was used to identify a systematic evolution of SBA‐15 characteristics. The addition of fluoride thereby promoted the effect already initiated by the hydrothermal aging. In general, a higher aging temperature, a longer dwell time, and a higher fluoride concentration leads to a decrease in the overall surface area, microporosity, and an increase in the mesopore diameter. For intact 2D SBA‐15, the hexagonal unit‐cell parameter increases linearly as a function of the mesopore diameter under those conditions, whereas for disordered 3D SBA‐15 the unit‐cell parameter is decoupled from the evolution of the other physical properties.

The use of aging conditions of 130 °C for 24 h or 0.05 equiv NH_4_F at 80 °C for 24 h both lead to structurally intact, uniform, and highly ordered 2D SBA‐15 materials. The increase in aging temperature, aging time, or fluoride concentration for one of the described set of conditions led to the formation of mesotunnels (samples with *S*_total_ of ≈570 m^2^ g^−1^) and the development of a 3D network based on the mesoporous connectivity (loss of mesoporous order). This is comprehensively demonstrated by the broadening of the pore size distribution and the decrease of ordered structures and the corresponding long‐range order (based on low‐angle XRD analysis). A distinct low‐angle contribution (0.45° to 0.65°) in the low‐angle XRD increased with increasing disorder of the corresponding sample. For purely 2D SBA‐15 samples, this feature could not be observed, thereby serving as a suitable descriptor for the 3D SBA‐15 formation. The disorder and mesotunnel formation were emphasized with progressing aging temperature, time, and fluoride concentration and caused the formation of amorphous and highly disordered silica structures (samples with *S*_total_<250 m^2^ g^−1^). This structural disintegration is additionally supported by TEM analysis. Overall, the impact of, particularly, fluoride on the silica structure was demonstrated to be significant, with respect to structural order.

A consequence of the disintegration was thereby demonstrated by using the conversion of isopropanol as a probe reaction for the chemical surface properties. A correlation between a change of the physical and chemical properties was evidenced. A higher amount of disordered silica fractions resulted in the formation of more active sites on the silica surface. The reason for the different reactivities is caused by various Lewis acid sites as identified by FTIR (H/D exchange using D_2_). The order in reactivity follows the exchange kinetic of H/D, being particularly pronounced for the NH_4_F‐treated samples. It is indicated that the loss in microporosity and structural integrity when using NH_4_F is a subsequent event additionally influencing the surface properties.

In summary, the modified synthesis conditions have a strong impact on the material's structure (transition of 2D and 3D pore network) and surface properties. These surface alterations directly influence the catalytic reactivity with distinct consequences on the applicability of 2D SBA‐15 or 3D SBA‐15 (interconnected mesopores) materials. Our results confirm the benefit of complementary techniques (bulk and surface specific), here of the isopropanol conversion reaction as a suitable test reaction for chemical surface reactivity, applied as characterization tools in general.

## Conflict of interest

The authors declare no conflict of interest.

## Supporting information

As a service to our authors and readers, this journal provides supporting information supplied by the authors. Such materials are peer reviewed and may be re‐organized for online delivery, but are not copy‐edited or typeset. Technical support issues arising from supporting information (other than missing files) should be addressed to the authors.

Supplementary
