# Supplementary material for: Transition from 2D to 3D SBA‐15 by High‐Temperature Fluoride Addition and its Impact on the Surface Reactivity Probed by Isopropanol Conversion
Source: Chemistry. 2020 Aug 6;26(50):11571–83. doi: 10.1002/chem.202001646 (PMC7540577; doi:10.1002/chem.202001646)
Supplement: Supplementary file 1 — Supplementary [file CHEM-26-11571-s001.pdf]

Chemistry–A European Journal

Supporting Information

Transition from 2D to 3D SBA-15 by High-Temperature Fluoride Addition and its Impact on the Surface Reactivity Probed by Isopropanol Conversion

Maximilian Lamoth,[a] Thomas Gries,[a] Frank Girgsdies,[a] Friedrich Seitz,[a] Maike Hashagen,[a] Frank Rosowski,[b, c] Robert Schlögl,[a, d] and Elias Frei*[a]

SUPPORTING INFORMATION

Table S1. Overview of SBA-15 samples including the FHI database sample numbers, the fitting errors for NLDFT method for pore size distribution from which the average pore diameter (D_p) is determined and the triple estimated standard deviation for the determination of hexagonal unit cell parameter a_0 from low angle XRD.

Sample label	FHI sample #	D_P [Å]	Fitting error [%]	a_0 [Å]	3*esd [Å]
LTA_12/80	24827	73.1	0.56	111.0	0.1
LTA_24/80	24836	75.9	0.59	111.9	0.1
LTA_84/80	24743	81.4	0.94	115.0	0.1
HTA_24/80	24883	75.9	0.57	111.6	0.1
HTA_24/100	24908	81.4	0.93	116.1	0.1
HTA_24/110	24920	87.8	0.97	119.0	0.1
HTA_24/120	24921	91.0	0.96	119.0	0.1
HTA_24/130	24922	94.2	1.16	119.9	0.1
HTA_24/140	24923	108.9	0.94	122.5	0.2
HTA_168/130	25235	108.8	0.82	120.0	0.3
HTA_312/130	25238	129.9	0.68	120.8	0.2
$HTAF_{0.05}$_24/80	28660	94.2	1.39	123.5	0.1
$HTAF_{0.10}$_24/80	28661	104.9	0.95	124.8	0.2
$HTAF_{0.15}$_24/80	28662	108.8	0.92	125.4	0.2
$HTAF_{0.20}$_24/80	28663	116.8	0.84	125.2	0.2
$HTAF_{0.05}$_24/110	28461	116.8	1.07	124.8	0.1
$HTAF_{0.10}$_24/110	26894	113.0	0.90	125.2	0.2
$HTAF_{0.15}$_24/110	26897	126.0	0.66	125.5	0.2
$HTAF_{0.05}$_168/110	28466	199.0	0.77	127.0	0.4
$HTAF_{0.10}$_168/110	26896	238.0	0.82	129.0	0.5
$HTAF_{0.15}$_168/110	28467	350.0	1.04	n/a	n/a
$HTAF_{0.05}$_24/130	26890	104.9	0.95	121.9	0.1
$HTAF_{0.10}$_24/130	26040	121.2	1.22	124.7	0.2
$HTAF_{0.20}$_24/130	28564	129.9	0.81	126.7	0.2
$HTAF_{0.25}$_24/130	28565	166.9	0.87	127.0	0.2
$HTAF_{0.05}$_168/130	26041	166.8	0.91	125.5	0.2
$HTAF_{0.10}$_168/130	26042	199.0	1.00	125.9	0.2
$HTAF_{0.20}$_168/130	26043	273.7	1.23	125.8	0.2

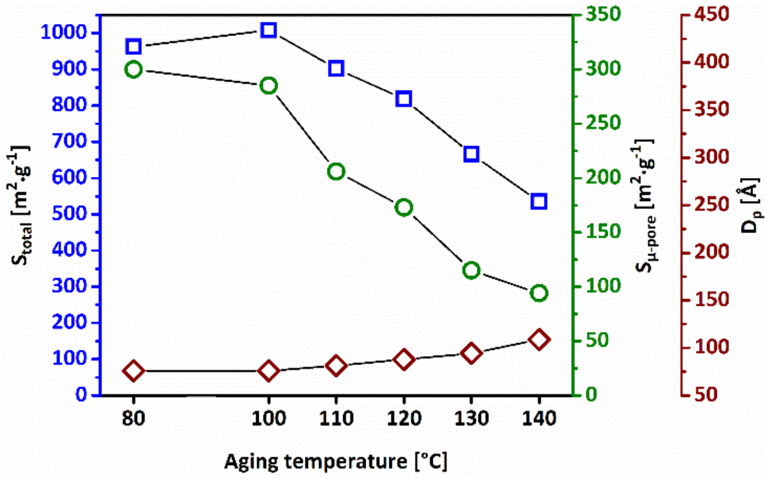

Figure S1. Total surface areas (S_{total}), micropore surface areas ($S_{\mu\text{-pore}}$) and mesopore diameters (D_p) of HTA SBA-15 aged at 80 – 140 °C for 24 h.

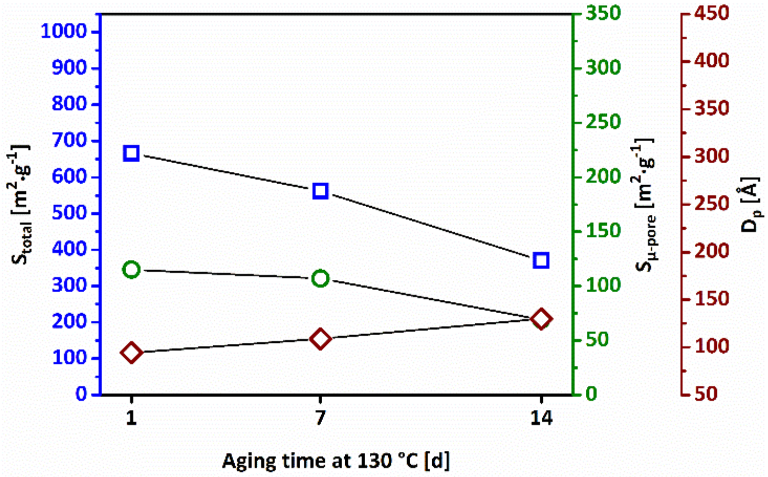

Figure S2. Total surface areas (S_{total}), micropore surface areas ($S_{\mu\text{-pore}}$) and mesopore diameters (D_p) of SBA-15 aged at 130 °C for 1, 7 and 14 d.

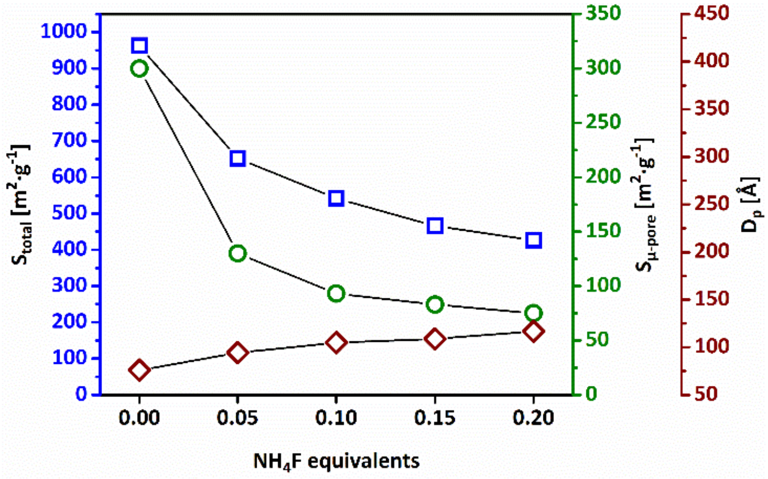

Figure S3. Total surface areas (S_{total}), micropore surface areas ($S_{\mu\text{-pore}}$) and mesopore diameters (D_p) of SBA-15 aged at 80 °C for 24 h with addition of different NH_4F equivalents of 0 – 0.20.

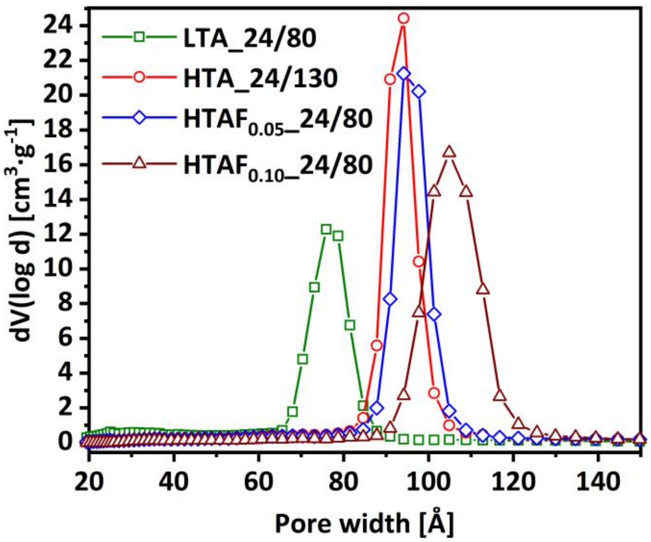

Figure S4. Pore size distributions of SBA-15 samples aged at 80 °C for 24 h (green squares), 130 °C for 24 h (red circles), 80 °C for 24 h with 0.05 eq NH_4F (blue diamonds) and 0.10 eq NH_4F (dark red triangles)

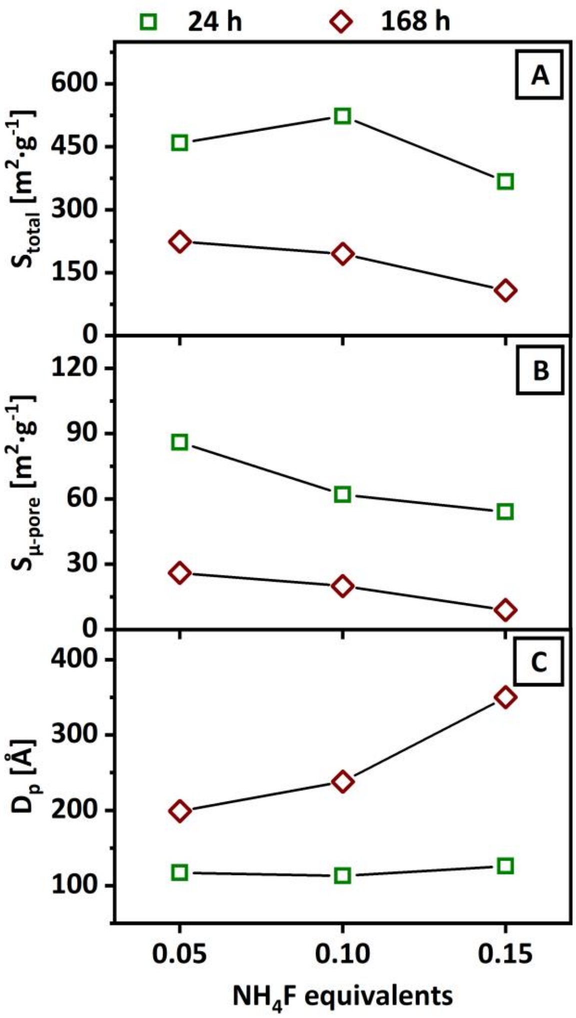

Figure S5. Total surface areas (S_{total}, A), micropore surface areas ($S_{\mu\text{-pore}}$, B) and mesopore diameters (D_p, C) of SBA-15 aged at 110 °C for 1 – 7 d as a function of the NH_4F addition of 0.05 – 0.15 equivalents.

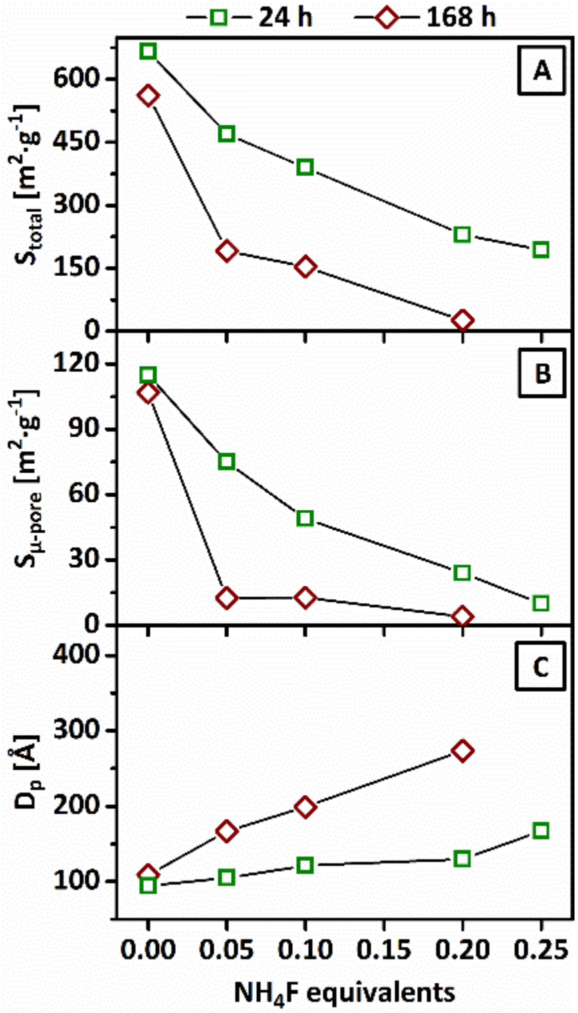

Figure S6. Total surface areas (S_{total}, A), micropore surface areas ($S_{\mu\text{-pore}}$, B) and mesopore diameters (D_p, C) of SBA-15 aged at 130 °C for 24 h (green squares) and 168 h (red diamonds) as a function of the NH_4F addition of 0 – 0.25 equivalents.

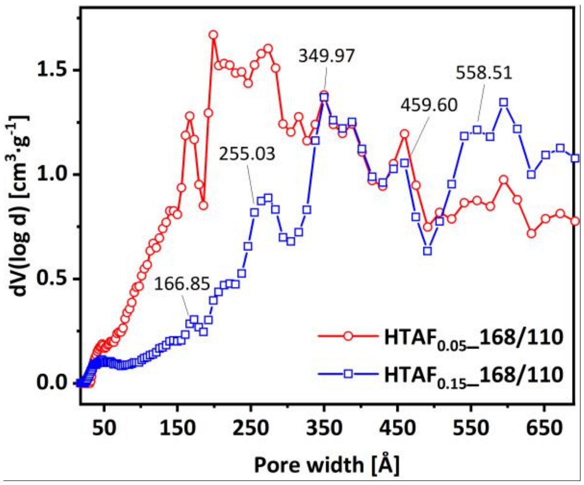

Figure S7. Pore size distribution for SBA-15 aged at 110 °C for 168 h with the addition of 0.05 eq (red) and 0.15 eq NH_4F (blue).

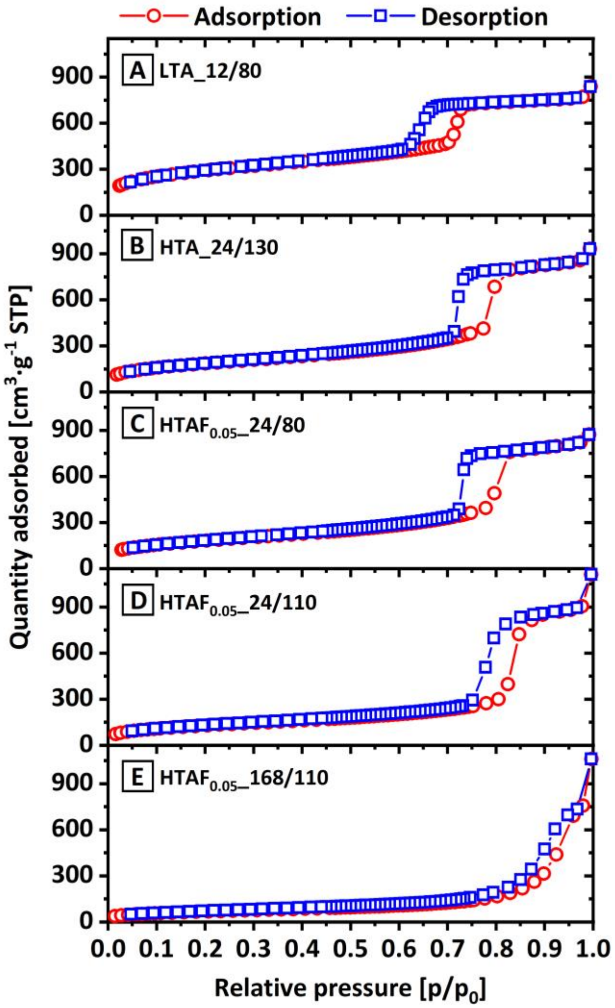

Figure S8. Full range N_2 physisorption isotherms with adsorption (circles, red) and desorption (squares, blue) for samples LTA_12/80 (A), LTA_24/80 (B), HTA_24/130 (C), $HTAF_{0.05}$_24/80 (D), $HTAF_{0.05}$_24/110 (E) and $HTAF_{0.05}$_168/110 (F).

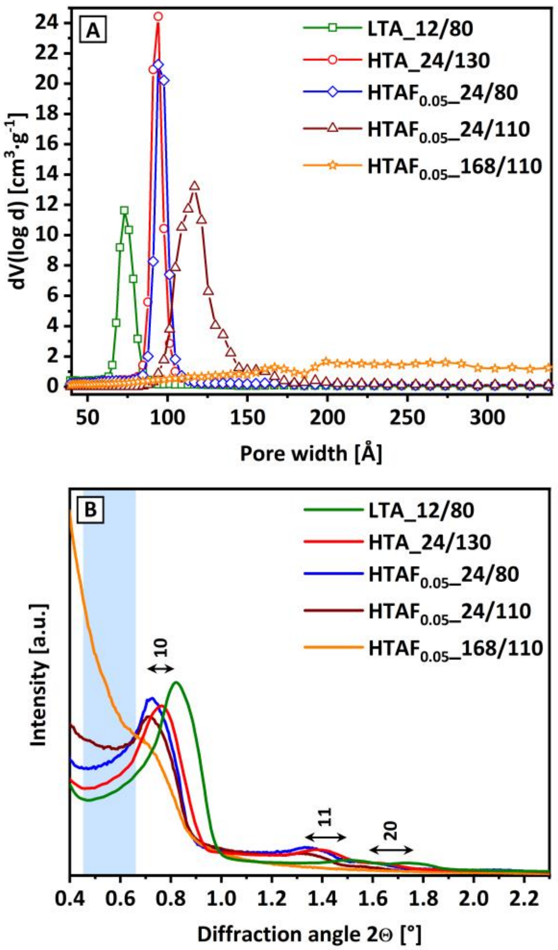

Figure S9. Pore size distributions (A) and low angle XRD (B) of samples aged at 80 °C for 12 h (green), aged at 130 °C for 24 h (red), and the addition of 0.05 eq NH_4F and aged at 80 °C for 24 h (blue), 110 °C for 24 h (wine) and 110 °C for 168 h (orange). The blue highlight (B) shows the appearance of a "disorder signal".

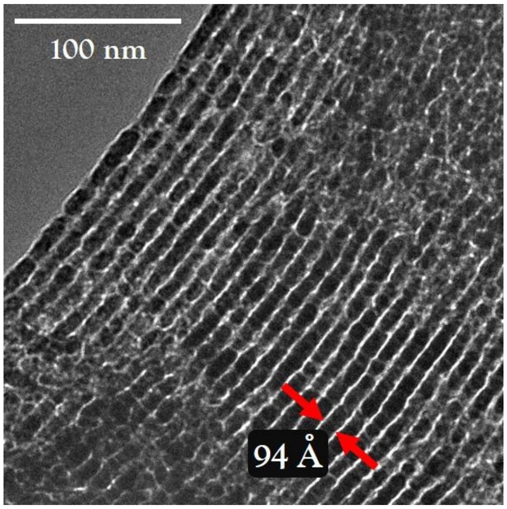

Figure S10. TEM analysis of sample $HTAF_{0.05}$_168/110 showing non-dominant regular silica structures.

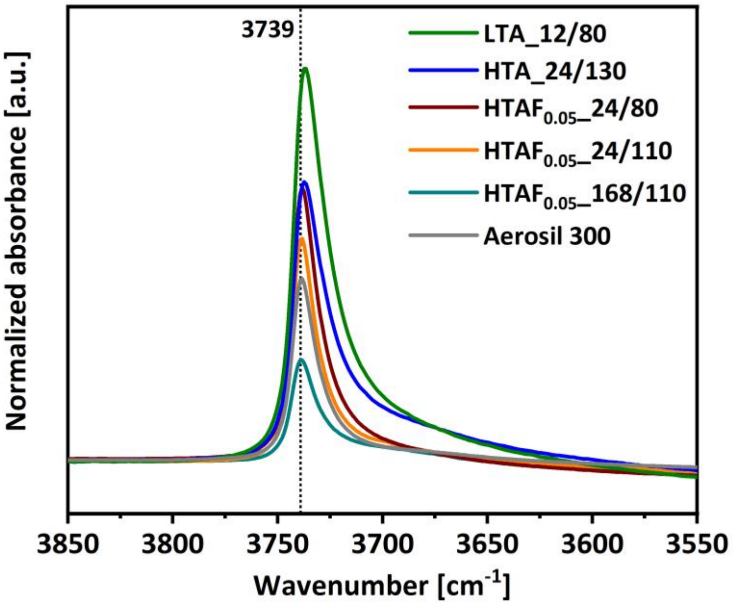

Figure S11. IR spectra of samples LTA_12/80 (green), HTA_24/130 (blue) and samples with NH_4F addition of 0.05 eq treated at 24/80 (wine), 24/110 (orange) and 72/110 (dark cyan). In addition, commercially available fumed silica is tested as reference (Aerosil, grey). Absorbance is normalized to area weight.

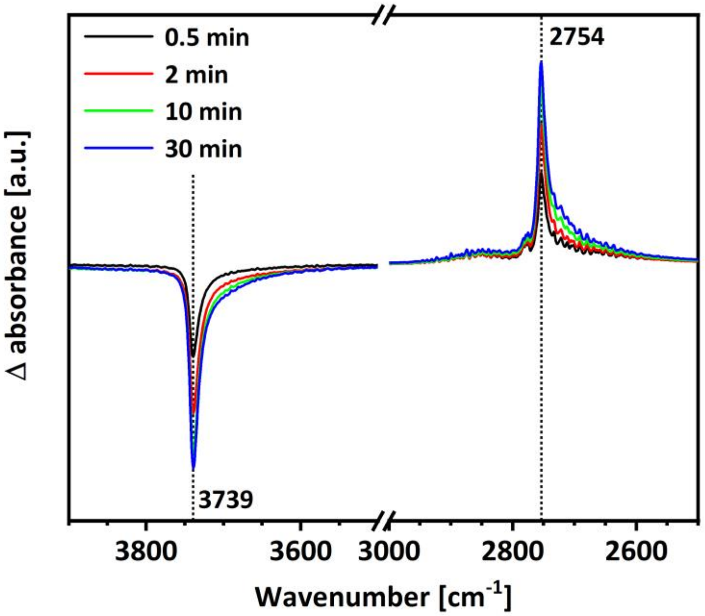

Figure S12. IR spectra of sample $HTAF_{0.05}$_168/110 after dosing 100 mbar D_2 at 400 °C. Spectra were recorded 0.5 (black), 2 (red), 10 (green) and 30 min (blue) after addition and are referenced against a spectrum taken immediately before addition.

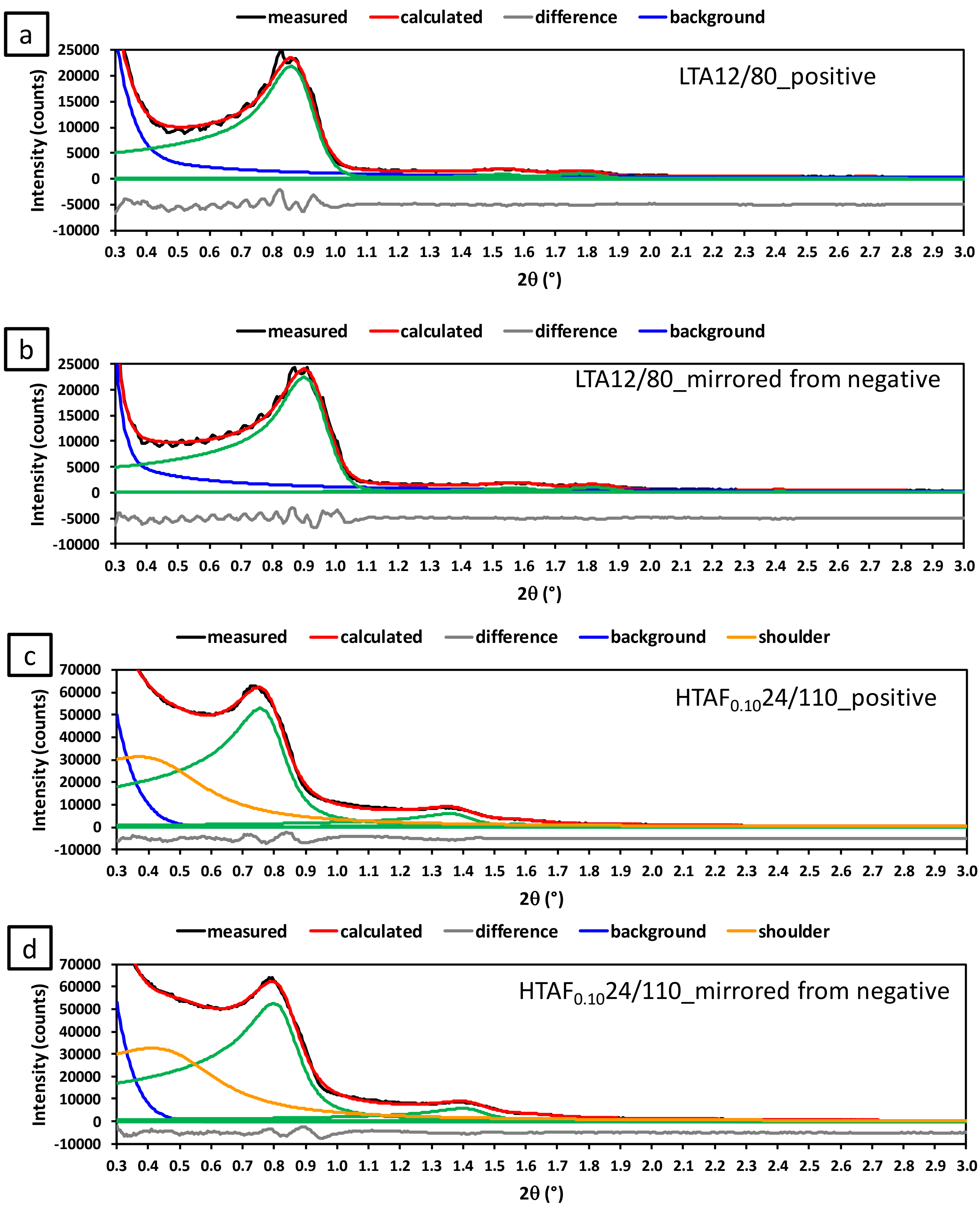

Figure S13. Diffraction patterns as example for the fitting procedure and the quality of the fits, respectively, from which the results in Table 1 were extracted. (a) and (b) show results of a sample with 2-D mesoporous character without shoulder for positive and negative 2θ values. The negative values are mirrored for comparison reasons into positive ones. (c) and (d) show an example for a sample with rather 3-D mesoporous character and a pronounced shoulder.
